# Measles in a Micronesian Community — King County, Washington, 2014

**Published:** 2014-09-12

**Authors:** Kristen Wendorf

**Affiliations:** 1 Epidemic Intelligence Service, CDC; 2 Public Health–Seattle and King County, Seattle, Washington

Measles is a highly contagious viral disease that can lead to complications and death. The United States achieved measles elimination (interruption of continuous transmission lasting ≥12 months) in 2000. Despite elimination, 592 measles cases were reported in the United States during January 1–August 22, 2014, the highest number since 1994 (1), primarily among unvaccinated travelers and their unvaccinated contacts. Measles remains endemic outside the Western Hemisphere, with outbreaks affecting communities in the Philippines, Vietnam, and China (1,2). An ongoing measles outbreak with approximately 350 measles cases and one death in the Federated States of Micronesia during January–July 2014 also has been reported (3).

On May 30, 2014, a child in King County, Washington, aged 4 years and unvaccinated against measles, developed a measles rash 4 days after returning home from 2 weeks in the Federated States of Micronesia. During the following 5 weeks, 14 additional measles cases (nine laboratory-confirmed B3 wild-type and five epidemiologically linked) were reported in King and Pierce counties. Patients were aged 5 months–48 years (median = 3 years). Two patients were too young to have been vaccinated against measles according to U.S. recommendations, nine were aged >12 months and unvaccinated against measles, three had received 1 dose of measles-containing vaccine, and one had received 2 doses. Twelve cases occurred in the local Micronesian community, in which many children and adults have no documentation of measles vaccination; during two community vaccination clinics early in the outbreak, 71% of the 267 community members who came to the clinic had no electronic or written vaccination record nor knowledge of previous measles vaccination. Large, loosely defined family structures pose challenges for case and contact investigations. Additional exposures occurred in medical facilities and workplaces; six patients visited more than one acute care facility for treatment while they were infectious.

Local public health officials conducted extensive community outreach (in collaboration with a Micronesian community liaison), contact tracing, and community-based vaccination clinics. Measles outbreaks are ongoing in the Federated States of Micronesia. The risk for acquiring measles during travel to the area is elevated, especially among unvaccinated persons.

This outbreak demonstrates the ease with which measles can be imported from a country with an ongoing outbreak and spread among a local population. These events also highlight the need for directed community outreach regarding the importance of routine vaccinations, including vaccination before travel. Health care providers should be vigilant for measles among persons with febrile rash illness returning from countries with ongoing measles transmission.
